# Multitype Bellman-Harris branching model provides biological predictors of early stages of adult hippocampal neurogenesis

**DOI:** 10.1186/s12918-017-0468-3

**Published:** 2017-10-03

**Authors:** Biao Li, Amanda Sierra, Juan Jose Deudero, Fatih Semerci, Andrew Laitman, Marek Kimmel, Mirjana Maletic-Savatic

**Affiliations:** 1 Departments of Bioengineering and Statistics, Rice University, Houston, Texas, 77005 USA; 2 Department of Pediatrics, Baylor College of Medicine, Houston, Texas, 77030 USA; 3 Jan and Dan Duncan Neurological Research Institute at Texas Children’s Hospital, Houston, Texas, 77030 USA; 4 Program in Developmental Biology, Baylor College of Medicine, Houston, Texas, 77030 USA; 5 Structural and Computational Biology and Molecular Biophysics Program, Baylor College of Medicine, Houston, Texas, 77030 USA; 6 Systems Engineering Group, Silesian University of Technology, Gliwice, 44–100 Poland; 7 Department of Neuroscience, Baylor College of Medicine, Houston, Texas, 77030 USA; 8 Department of Statistics, Rice University, Houston, Texas, 77005 USA

**Keywords:** Hippocampus, Adult neurogenesis, Apoptosis, Computational modeling, Multitype Bellman-Harris branching process

## Abstract

**Background:**

Adult hippocampal neurogenesis, the process of formation of new neurons, occurs throughout life in the hippocampus. New neurons have been associated with learning and memory as well as mood control, and impaired neurogenesis has been linked to depression, schizophrenia, autism and cognitive decline during aging. Thus, understanding the biological properties of adult neurogenesis has important implications for human health. Computational models of neurogenesis have attempted to derive biologically relevant knowledge, hard to achieve using experimentation. However, the majority of the computational studies have predominantly focused on the late stages of neurogenesis, when newborn neurons integrate into hippocampal circuitry. Little is known about the early stages that regulate proliferation, differentiation, and survival of neural stem cells and their immediate progeny.

**Results:**

Here, based on the branching process theory and biological evidence, we developed a computational model that represents the early stage hippocampal neurogenic cascade and allows prediction of the overall efficiency of neurogenesis in both normal and diseased conditions. Using this stochastic model with a simulation program, we derived the equilibrium distribution of cell population and simulated the progression of the neurogenic cascade. Using BrdU pulse-and-chase experiment to label proliferating cells and their progeny in vivo, we quantified labeled newborn cells and fit the model on the experimental data. Our simulation results reveal unknown but meaningful biological parameters, among which the most critical ones are apoptotic rates at different stages of the neurogenic cascade: apoptotic rates reach maximum at the stage of neuroblasts; the probability of neuroprogenitor cell renewal is low; the neuroblast stage has the highest temporal variance within the cell types of the neurogenic cascade, while the apoptotic stage is short.

**Conclusion:**

At a practical level, the stochastic model and simulation framework we developed will enable us to predict overall efficiency of hippocampal neurogenesis in both normal and diseased conditions. It can also generate predictions of the behavior of the neurogenic system under perturbations such as increase or decrease of apoptosis due to disease or treatment.

## Background

Adult neurogenesis generates new neurons throughout life in two distinct regions of the mammalian brain: the subventricular zone, involved in olfactory processes, and the sub-granular zone (SGZ) of the dentate gyrus [1–4], where new neurons have been associated with learning and memory as well as mood control [5–7]. The addition of new neurons is not merely static or restorative; it constitutes an adaptive response to the animal’s environment and/or its internal state. For example, hippocampal neurogenesis can be regulated both positively and negatively by external stimuli, such as learning [8], exercise [9], environment [10] and stress [11], as well as pathological states such as epilepsy [12–16], drug addiction [17–19], depression [20–22] and schizophrenia [23, 24]. Thus, adult neurogenesis represents another means, apart from molecular, synaptic, or morphological changes of an individual cell, to alter the functional circuitry depending on the demand. However, despite a significant functional relevance of this form of whole-cell plasticity, little is known about the processes that regulate it.

**Fig. 1:**
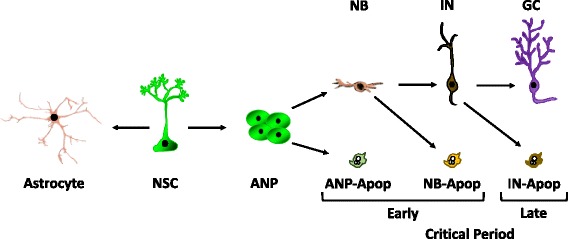
Hippocampal neurogenic niche produces new neurons through a cascade of different cell types. Neural stem cells (NSCs) provide a basal level influx of new amplifying neuroprogenitors (ANPs) through asymmetric divisions. Newborn ANPs divide several times but only some of them survive to differentiate into the early neuroblast (NB). As these cells continue to differentiate into immature neurons (IN), their numbers are reduced further. In the end, only a few mature neurons, so called granule cells (GC), are produced. Throughout the neurogenic cascade, the different cell types undergo apoptosis (Apop). The apoptotic cells live for a short period of time because they are rapidly phagocytosed and degraded by the resident microglia

During physiological conditions, adult neurogenesis maintains a steady-state. At any given moment, neural stem and progenitor cells (NPCs) may undergo one of three possible fates – they proliferate, producing more of identical daughter cells; they differentiate, giving rise to immature neurons called neuroblasts; or they die [25–28] (Fig. 1). It is believed that the basal rate of neurogenesis is genetically determined [29], but the mechanisms that govern it under various physiological and pathological stimuli are poorly understood. Most research on the neurogenic regulatory mechanisms has centered on the factors that regulate NPC proliferation and integration of newborn neurons into the dentate circuitry during the late stages of neurogenic cascade [30, 31]. However, early stages of neurogenesis are very complex, as mechanisms that determine cell proliferation, differentiation, and death are active at the same time. Further, the influence of the newborn cell death on adult neurogenesis is not known, even though it has been established that the majority of newborn cells in the adult dentate SGZ die during the first week of life, presumably undergoing apoptosis [32]. Newborn cell apoptosis may also be important for spatial learning [33], a hippocampal-dependent task suggested to require neurogenesis [34]. Thus, understanding the early stages of neurogenesis is critical if we are to manipulate this process to enhance the number of viable newborn neurons as treatment modalities.

As experimental studies of such a complex system require years of work, several groups have used computational tools to aid discovery and guide biological experimentation. Most of the existing models, however, focus on the late stages of neurogenesis and aim to understand the effects of new neuron incorporation into the dentate gyrus. These models have shown that new granule cells participate in pattern separation, avoiding interference between memories while older ones are not greatly disturbed [35–37]. However, existing models have not addressed all the processes that occur throughout the neurogenic cascade, specifically all three possible fates of cells that are part of the neurogenic cascade - proliferation, differentiation and cell death. Hence, here we propose a comprehensive computational model of all stages of neurogenic cascade, including transition, proliferation, differentiation and survival of newborn cells from the NPC stage to the stage of a matured neuron. To model early stages of neurogenesis, we use the theory of branching processes [38]. In a hierarchical system such as that formed by proliferating and differentiating ANPs, the theory allows to formulate explicit analytic solutions in the terms of multiple but finite-order convolutions of distributions of transit times through different phases of cell cycle. This latter feature makes modeling particularly transparent, and allows avoiding purely numerical simulations. Branching process theory can be traced to the social scientists in the 19th century studying the extinction of family lines. From that time on, a large number of biological problems have been modeled by branching processes, particularly in the analysis of evolutionary cell population and population genetics. For example, during the evolution of a population of some reproducing particles, each particle lives for a lifetime, independently of the others, and produces a random number of new offspring. If each particle lives for a constant unit of time and produces progeny upon death, then the process is called a Galton-Watson branching process. If each particle has an exponentially distributed lifetime independent of the offspring distribution, then the process is called a Markov continuous-time branching process. If the lifetime of each particle is a random variable with an arbitrary distribution, independent of lifetimes of the offspring, then this process is named an age-dependent (Bellman-Harris) branching process [38]. Here, using the Multitype Bellman-Harris branching model, we provide for the first time estimates of the early stages of the neurogenic cascade, focusing on the apoptosis and transit times of cells, from birth to incorporation into the hippocampal circuitry.

## Methods

### Experimental methods

#### Animals

Wild-type (C57BL/6) or transgenic *Nestin*-CFPnuc mice, in which CFP is expressed in the nuclei of both neural stem cells (NSCs) and ANPs [21], were used. All mouse studies were approved by the Baylor College of Medicine Institutional Animal Care and Use Committee and performed in accordance with institutional and federal guidelines. Unless otherwise stated, animals were 1 month old.

#### Cell labeling with Bromodeoxyuridine (BrdU)

**Fig. 2:**
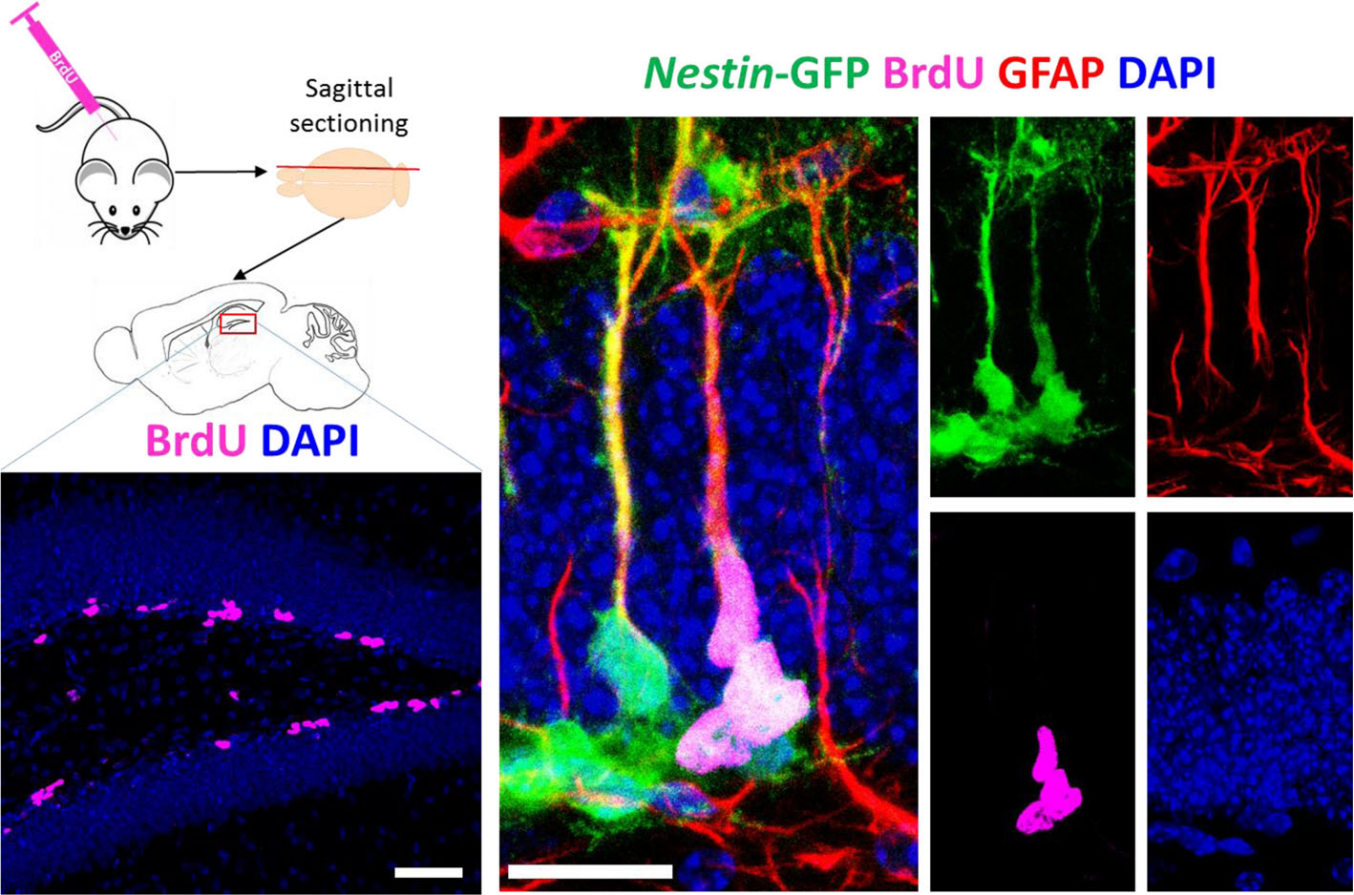
BrdU labels dividing cells in S-phase. 5’-Bromo-2’-deoxyuridine (BrdU) incorporates into the newly synthesized DNA during cell division and can be detected with specific antibodies. BrdU is injected intraperitoneally and the animal is sacrificed at a given time. The brain is isolated and sectioned sagittally for the best visualization of the dentate gyrus. The representative low-magnification image of the dentate gyrus has granule cells labeled by DAPI (blue) and dividing cells labeled by BrdU (pink) (scale bar=20um). In the high magnification confocal micrographs, NSCs and their progeny, ANPs, express Nestin-GFP (green). In addition, NSCs express GFAP (red), while ANPs do not. BrdU-labeled dividing cells are in pink. Scale bar = 50um

**Fig. 3:**
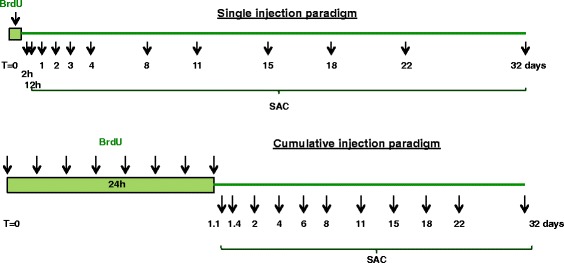
Experimental flow-chart of single and cumulative BrdU labeling. Arrows pointing to the green box indicate time points of BrdU injection in two experimental designs, while lower arrows pointing to numbers (hours (h) or days) stand for time points when animals were sacrificed (SAC). For the purpose of modeling and computation, we used single injection paradigm, while data for the apoptotic experiments were derived from the cumulative BrdU paradigm

When studying neurogenesis, the most accepted method to estimate the net effect of the neurogenic cascade is to use BrdU, which labels cells in S phase of the cell cycle, to trace proliferation and differentiation (Fig. 2). BrdU is injected and during the circulating 15 min time, it gets incorporated into a proliferating DNA. Over the course of the neurogenesis, the BrdU can be traced in cells that are the lineage of the initial proliferating cell. BrdU labeling can be done as a single or cumulative injection paradigm (Fig. 3). In single labeling experiments, animals were injected with 250 mg/kg BrdU at *t*=0. In cumulative labeling experiment, performed to obtain the highest yield of the apoptotic cells, animals were injected with 150 mg/kg BrdU every 3 h in the first 24 h, totally 9 injections including the one at *t*=0. Animals were then sacrificed at different time points, when the total number of BrdU cell as well as the percentage of cells in each stage of the cascade was quantified.

#### Histology

Mice were transcardially perfused with phosphate buffer saline (PBS) followed by 4% paraformaldehyde (PFA). The brains were dissected out submerged into 4% PFA for 4 h at room temperature (RT) and sectioned using a vibratome. For immunofluorescence, free-floating sections were immunolabeled according to conventional procedures. The brains were dissected out, and then transferred to a cryoprotectant solution (30% sucrose, 30% ethylene glycol in PBS) and stored at -20 °C. Once brains were well cryoprotected, six series of 50 *μ*m lateral sections were collected using a vibratome. A full series of free-floating sections were immunostained following conventional procedures [39]. Briefly, all washes and incubations were done in PBS containing 3% bovine serum albumin (BSA; Sigma-Aldrich) and 0.3% Triton X-100 (Sigma-Aldrich). An antigen retrieval step (2 N HCl, 15 min, 37 °C) for BrdU detection was performed, followed by extensive washes with borate buffer (0.1 M). Sections were pre-incubated in PBS containing 3% BSA, 5% normal goat serum (Vector Labs) and 0.3% Triton X-100 for 2 h at RT, followed by overnight incubation with primary antibodies (see below) at 4 °C. After extensive washing, sections were incubated with the appropriate secondary antibody conjugated with Alexa 488 (Molecular Probes), Rhodamine Red-X and Cyanine 5 (Jackson Immunoresearch) together with DAPI (5 *μ*g/mL, Sigma-Aldrich) for 2 h at RT. They were then washed, and mounted on slides with Fluorescent Mounting Medium (Dako). The following primary antibodies were used: BrdU (1:400, Accurate) to detect proliferating cells in S phase; DCX (Cell Signaling, 1:200) to detect neuroblasts/immature neurons; GFAP (1:1,000, Sigma-Aldrich) to detect primary neural stem cells (NSCs) and distinguish them from the ANPs (GFAP-); NeuN (1:1,000, Chemicon) to detect mature neurons of the dentate gyrus, granule cells.

#### Confocal Microscopy

Sections were imaged with a Zeiss LSM or a Leica SP5 confocal microscope. The number of apoptotic cells and/or BrdU+ cells per z-stack was estimated via the optical dissector method [39]. Blind analysis was performed with AxioVision 4.5 (Zeiss) or LAS AF Lite (Leica). Two-three 20 *μ*m z-stacks (consisting of 30 optical slices of 0.8 *μ*m thickness) were obtained from every section. The number of cells was evaluated as a function of the volume of the SGZ, defined as the SGZ length in the image multiplied by an optical thickness of 20 *μ*m and a height of 20 *μ*m (which we defined in these experiments as a layer of cells expanding 5 *μ*m into the hilus and 15 *μ*m into the granular layer), then extrapolated to the volume spanned by the SGZ in the hippocampus.

### Computational methods

#### Cell transit times

Cell transit time is defined as the duration time of a cell spent in the phase or stage before it transits into the next phase or stage. Instead of the commonly used exponential distributions, we use shifted gamma distributions of the transit times [40] to model the cell transit times through phases G1, S and G2M, as well as the lifetime of cells. Advantage of these is the ability to independently specify the minimum transit times, mean transit times, and variances of transit times. Among other, this allows avoiding occurrence of cells that live for an indefinitely short period of time with certain probability. Such distribution has three parameters, (*k,s,v*), with its probability density function given as *f*(*x*|*k,s,v*)=(*x*−*v*)^*k*−1^ *e* ^−(*x*−*v*)/*s*^/(*Γ*(*k*)*s* ^*k*^), where *k* is the shape parameter, *s* is the scale parameter and *v* is the shift value (minimum duration), and *Γ*(*k*) is Euler gamma function [40]. Cells in different cell cycle phases are assigned shifted gamma distributions with different sets of parameters.

#### Classification of cell stages

The hypothesized neurogenic cascade consists of i) multiple cell types as different states, and ii) probabilities of cells progressing from one type to another as transition rates between states. Our model includes the following cell types: primary NSCs, ANPs, neuroblast/immature neurons (NBs), granule cell neurons (GCs) and apoptotic cells (Apop).

#### NSC (neural stem cell)

NSCs provide the ultimate influx of newborn ANPs, which massively proliferate to drive the entire cell population to produce mature granule cells. The majority of NSCs are quiescent while activated ones divide asymmetrically to enrich the pool of newborn ANPs. The baseline NSC-ANP influx can be modeled as a homogeneous Poisson Process. Even though the influx rate may change as the animal ages, we assume it to be fixed at 1-month-of-age, as all our experimental data are acquired at this age. To quantify the number of proliferating NSCs, we apply BrdU pulse-and-chase experiment, where 150 mg/kg BrdU is given intraperitoneally to the mouse. Its half life is about 15 min; thus all activated NSCs that are in the S phase are labeled. We call these BrdU+ NSCs - labeled NSCs. Encinas et al., (2011) indicate that each newly activated NSCs proliferates three times to produce three ANP progeny asymetrically and eventually becomes an astrocyte.

#### ANP (amplifying neuroprogenitor)

Each newborn ANP proliferates several times (~2.45 on average, estimated by Encinas et al., 2011). After the cell divides for a minimum number of times (≥1), it can either keep proliferating, differentiate into a neuroblast, or die. We model ANP progression by specifying minimal/maximum number of divisions and renewal probability (*p*), which denotes the probability of an ANP continuing to proliferate after finishing its minimal number of divisions. If an ANP chooses to proliferate, it enters typical cell cycle phases of G_1_, S and G_2_M; otherwise, it becomes a non-proliferating ANP which may choose to differentiate to neuroblast (NB) by entering ANP-NB transition state or commit programmed cell death (apoptosis or Apop) by entering the ANP-Apop transition state. Note: We assume that cells that are in either ANP-NB or ANP-Apop stage are still ANPs, but non-proliferating.

#### NB (neuroblast)

NBs are non-proliferating cells that are differentiated from ANPs. The celll duration in NB stage is relatively long (2–10 days) and eventually, each NB may choose to differentiate to immature neuron or enter apoptosis.

#### (immature neuron)

Similarly as NB, any immature neuron lives for a period of duration time and at the end, differentiates into a mature neuron (granule cell) or dies.

#### GC (granule cell)

GCs are fully differentiated and mature neurons of the dentate gyrus that remain in dentate gyrus to form neuronal connections with existing neurons of the dentate and hippocampal circuitry. Once a GC is formed, it cannot die or differentiate anymore.

#### ANP-NB

This is an intermediate state of transition time from a non-proliferating ANP to a NB, where the duration is modeled in the same way as duration of any other cell type, as a shifted-gamma distribution.

#### ANP-Apop

Is an intermediate state of transition time for a non-proliferating ANP to become an apoptotic cell.

#### Apop (apoptotic cell)

We assume that apoptosis may occur at the end of any cell stage (G_1_, S, G_2_M, NB, IN) along the neurogenic cascade, except for the granule cells. After BrdU pulse, all cells that are dividing and in S phase will be labeled. From the observation of apoptotic-BrdU cell labeling curve, there are no labeled apoptotic cells observed at either 2 h or 12 h (Sierra et al., 2010) (Table 1), indicating that proliferating NSCs and their first progeny do not undergo apoptosis or that the removal of the apoptotic cells at these times is so fast that it escapes detection. The estimated duration of ANP_G_2_M phase is about 2 h [41]. A proportion of newly labeled cells that are in their final allowed division and also transiting from S to G_2_M would be captured by BrdU. These observations can indicate the existence of ANP-Apop stage, otherwise the apoptotic-BrdU cells should be seen at 2 h or 12 h after BrdU injection. Also, they imply that the apoptotic rate of cells in either S or G_2_M phase is close to 0; otherwise, cells that are labeled in late S phases can enter cell death immediately after BrdU injection while approaching the end of S or G_2_M phases.

**Table 1:** Model parameters that can be and those that cannot be estimated experimentally

Experimentally estimable	Difficult to determine by experiments
Average duration times	Intensity of NSC →ANP influx
Cell population size	Apoptotic rate at each stage
	ANP renewal probability
	Possible number of ANP divisions
	Shapes of distributions of durations
	Minimum durations

### Model assumptions

- The process of proliferation and maturation is driven by a steady influx of generation 1 ANPs (ANP1).
- Arriving ANP1 cells enter the G1 phase of the cell cycle
- Subsequently, the ANP1 cells proceed through G1, S and G2M phases before they split into two ANP2 cells.
- Each ANP2 cell proceeds through the G1, S and G2M phases before it splits into two cells, each of which may become a NB or a ANP3 cell.
- Each ANP3 cell proceeds through the G1, S and G2M phases before it splits into two cells, each of which becomes a NB.
- NBs exist for the time needed for them to become neurons.
- At each cell cycle phase, cells may enter apoptosis.
- Apoptotic cells are quickly engulfed by MG and eliminated.
- The transit times through the G1 phase is exponentially distributed with expected value T_1_, whereas transit times through phases S and G2M are deterministic equal to *T*_2_ and *T*_2_ respectively.
- The lifetime of the ANP is exponentially distributed with expected value *T*_4_, whereas the lifetime of the NB is exponentially distributed with expected value *T*_5_.

We denote *a* and *b* as the minimum and maximum number of divisions of each newborn ANP, where *a* is the required minimum number of divisions and *b* is the maximum allowed number of divisions. We further denote *p* as the renewal probability of each ANP (probability of proliferating after dividing *a* times) and denote *X* as the random variable of number of progeny produced by each new born ANP. Therefore, we obtain *P*(*X*=2^*a*^)=1−*p*, *P*(*X*=2^*a*+*i*^)=*p* ^*i*^(1−*p*), for 1≤*i*≤*b*−*a*−1 and *P*(*X*=2^*b*^)=*p* ^*b*−*a*^, and $E(X)=2^{a}(1-p)+\sum _{i=1}^{b-a-1}p^{i}(1-p)$ 2^*a*+*i*^+2^*b*^ *p* ^*b*−*a*^. For *a*<*b*, the expected number of ANP divisions can be derived as *log*_2_ *E*(*X*). If *a*=*b*, the expected number of division is *a*.

#### Modeling of the neurogenic cascade using the Bellman-Harris branching process

Our goal was to build a model of the early stage neurogenesis, from NSCs to newborn neurons, considering the factors influencing the ANP fate selection and cell death rates, such as transition to a cell cycle, number of divisions before differentiation into NB and probability of cell death at each step. We chose Multitype Bellman-Harris branching process to model the neurogenic cell population and resulting BrdU labeling curves. Bellman-Harris process is frequently employed to model proliferation of systems of biological cells [38], and in our model, it is necessary to distinguish cells in different cell cycle phases.

**Fig. 4:**
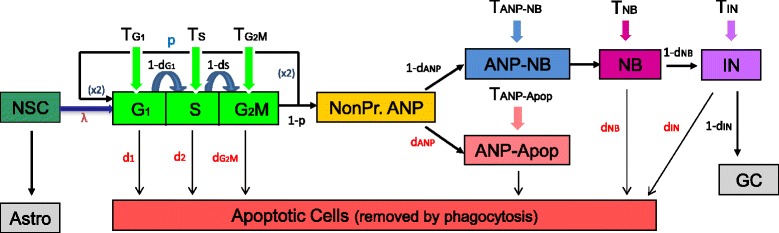
Hierarchical structure of the neurogenic cascade modeled by the Multitype Bellman-Harris branching process with different cell types as different compartments. Consider a collection of particles of *I* types, which proliferate according to the following rules: At time *t*=0, an ancestor particle of type *i* is born, which lives for a random time *τ* with cumulative distribution function (cdf) *T*_*i*_ and upon death, it produces a random number of progeny of all types, described by a vector (*X*_1_,...,*X*_*I*_) with multivariate probability generating function *h*_*i*_(*s*_1_,...,*s*_*I*_) At time *t*=*τ*, each first-generation progeny particle of type *j* lives for a random time with cumulative distribution function (cdf) *T*_*j*_ and upon death, produces a random number of progeny of all types, described by vector of multivariate pgf *h*_*j*_(*s*_1_,...,*s*_*I*_), independently of other progeny particles. The cycle of life, death and progeny production is repeated indefinitely by each generation of particles. ʎ= intensity of influx of new ANPs from NSCs, *X*_2_ = cell doubling rate, *D*_*i*_= cell death rate of the cell *i*, *p* = renewal probability of ANPs, *T*_*i*_= duration time of cell in stage *i*. G1, S, G2M = stages of cell cycle. NonPr ANP = non-proliferating ANP. Astro = astrocyte

The structure of the model, constructed based on our experimental observations [32], is presented in Fig. 4. We model the hierarchical structure with transition probabilities of cells from a stage to the next possible stage. Thus, cell death rates for different cell phases were modeled by the corresponding transit probabilities into apoptosis, where symbol *d*_*i*_ denotes the cell death rate of the cell type *i*.

If we denote the multivariate pgf of number of particles of all types present in the process initiated by an ancestor of type *i* with *F*_*i*_(*s,t*), we obtain the Bellman-Harris integral equation for this scenario as

$$F_{i}(s,t)=\int_{0}^{t}h_{i}[F(s,t-\tau)]d_{\tau}T_{i}(\tau)+s_{i}[1-T_{i}(t)] $$

Differentiating both sides of the equation with respect to *s*_*j*_ and setting *s*_1_=*s*_2_=...=*s*_*I*_=1, we may obtain the following equation for the matrix *M*(*t*)=[*M*_*ij*_(*t*)], where *M*_*ij*_(*t*) is the expected number of particles of type *j* at time *t*, in the process initiated by the ancestor particle of type *i* at time 0

$$M_{ij}(t)=\int_{0}^{t}\sum\limits_{k=1}^{I}m_{ik}M_{kj}(t-\tau)d_{\tau}T_{i}(\tau)+\delta_{ij}[1-T_{i}(t)] $$

where *m*_*ij*_(*t*) is the expected number of progeny of type *j* of a particle of type *i*, *δ*_*ij*_=1, if *i*=*j*, otherwise, *δ*_*ij*_=0.

The convolution of functions is defined by the following Lebesgue-Stieltjes integral if *A*(*t*) and *B*(*t*) are two right-continuous functions with locally bounded variation on [0,*∞*)

$$A(t)*B(t)=\int_{0}^{t}A(t-\tau)d_{\tau}B(\tau) $$

Using the convolution notation, the above equation can be expressed as

$$M_{ij}(t)=\sum_{k=1}^{I}m_{ik}M_{kj}(t)*T_{i}(t)+\delta_{ij}[\!1-T_{i}(t)] $$

In the matrix notation, we obtain

$$M(t)=T(t)*[\!mM(t)]+[\!I-T(t)] $$

where *I* is the identity matrix and *G*=*diag*(*G*_1_,...,*G*_*I*_). This is a renewal-type equation, which has a unique solution of locally bounded variation if *G*(0)=0, expressed by the infinite series

1 $$ M=\sum\limits_{k=0}^{\infty}(Tm)^{*k}*(I-T)  $$

and it yields the fundamental solution of the mathematical modeling of the neurogenic cascade as a matrix function of time for the number of cells of each type. Here, *M*_*ij*_(*t*) is the expected number of cells of type *j* at time *t*, in the process initiated by an ancestor cell of type *i* at time 0, *T*=*diag*(*T*_1_,...,*T*_*I*_) denotes the diagonal matrix with the distribution function of lifetime (or duration) *T*_*i*_ of each cell *i*, *m* is the transition matrix and *m*_*ij*_(*t*) is the expected number of progeny of cell of type *j* produced by a cell of type *i*, obtained via the multivariate pgf of numbers of progeny produced by the type *i* cell, and *I* is the identity matrix.

Based on the experimental observation and model assumptions, we have the transition matrix *m* as (e.g. when minimum/maximum number of ANP divisions are 1 and 3, respectively)

2 $$\begin{array}{@{}rcl@{}} \begin{array}{ccccccccccccccc} & \mathrm{G}_{1} & \mathrm{S} & \mathrm{G_{2}M} & \mathrm{G}_{1} & \mathrm{S} & \mathrm{G_{2}M} & \mathrm{G}_{1} & \mathrm{S} & \mathrm{G_{2}M} & \mathrm{A-N} & \text{NB} & \text{IN} & \mathrm{A-A} & \text{Apop}\\ \mathrm{G_{1}(1)} & 0 & \bar{d}_{G1} & 0 & 0 & 0 & 0 & 0 & 0 & 0 & 0 & 0 & 0 & 0 & d_{G1}\\ \mathrm{S(1)} & 0 & 0 & \bar{d_{S}} & 0 & 0 & 0 & 0 & 0 & 0 & 0 & 0 & 0 & 0 & d_{S}\\ \mathrm{G_{2}M(1)} & 0 & 0 & 0 & 2p\bar{d}_{G2M} & 0 & 0 & 0 & 0 & 0 & x^{*} & 0 & 0 & y^{**} & d_{G2M}\\ \mathrm{G_{1}(2)} & 0 & 0 & 0 & 0 & \bar{d}_{G1} & 0 & 0 & 0 & 0 & 0 & 0 & 0 & 0 & d_{G1}\\ \mathrm{S(2)} & 0 & 0 & 0 & 0 & 0 & \bar{d}_{S} & 0 & 0 & 0 & 0 & 0 & 0 & 0 & d_{S}\\ \mathrm{G_{2}M(2)} & 0 & 0 & 0 & 0 & 0 & 0 & 2p\bar{d}_{G2M} & 0 & 0 & x^{*} & 0 & 0 & y^{**} & d_{G2M}\\ \mathrm{G_{1}(3)} & 0 & 0 & 0 & 0 & 0 & 0 & 0 & \bar{d}_{G1} & 0 & 0 & 0 & 0 & 0 & d_{G1}\\ \mathrm{S(3)} & 0 & 0 & 0 & 0 & 0 & 0 & 0 & 0 & \bar{d}_{S} & 0 & 0 & 0 & 0 & d_{S}\\ \mathrm{G_{2}M(3)} & 0 & 0 & 0 & 0 & 0 & 0 & 0 & 0 & 0 & z^{*} & 0 & 0 & w^{**} & d_{G2M}\\ \mathrm{A-N} & 0 & 0 & 0 & 0 & 0 & 0 & 0 & 0 & 0 & 0 & 1 & 0 & 0 & 0\\ \text{NB} & 0 & 0 & 0 & 0 & 0 & 0 & 0 & 0 & 0 & 0 & 0 & \bar{d}_{NB} & 0 & d_{NB}\\ \text{IN} & 0 & 0 & 0 & 0 & 0 & 0 & 0 & 0 & 0 & 0 & 0 & 0 & 0 & d_{IN}\\ \mathrm{A-A} & 0 & 0 & 0 & 0 & 0 & 0 & 0 & 0 & 0 & 0 & 0 & 0 & 0 & 1\\ \text{Apop} & 0 & 0 & 0 & 0 & 0 & 0 & 0 & 0 & 0 & 0 & 0 & 0 & 0 & 0 \end{array} \end{array} $$

where A-N represents ANP-NB stage, A-A is for ANP-Apop stage, $\overline {d_{i}}=1-d_{i}$, $x^{*}=2\overline {d}_{G_{2}M}(1-p)\overline {d}_{ANP}$, *y* ^∗∗^=$2\overline {d}_{G_{2}M}(1-p)d_{ANP}$, $z^{*}=2\overline {d}_{G_{2}M}\overline {d}_{ANP}$ and $w^{**}=2\overline {d}_{G_{2}M}$ *d*_*ANP*_ (*d*_*ANP*_ is the cell death rate of non-proliferating ANPs).

Furthermore, to model the NSC to ANP influx, we assume that any ‘arrival’ of a new ANP is independent of all previous ‘arrivals’ and the number of new ANPs arrived during a period of time is only dependent on the length of that period times the intensity of the influx, *λ*. Thus, such process is a Poisson process with intensity parameter *λ*, and the probability that the number of new ANPs arrived during a time unit (*u*) being equal to *n* is expressed as

$$P[N(t+u)-N(t)=n]=\frac{e^{-\lambda u}(\lambda u)^{n}}{n!},\;n=0,1,... $$

where *N*(*t*) is the number of new ANPs arrived before time *t* and *N*(*t*+*u*) is the number of the new ANPs arrived until time *t*+*u*.

#### Modeling and simulation of cell labeling curves

The experimental data for our computational modeling have been obtained in three independent sets of time-course labeling experiments of pulse BrdU injection, with derived curves of total and partial cell counts at different times of measurements: 1) Single BrdU pulse-and-chase was used to quantify total BrdU+ cell populations over a 32 day period and total number of apoptotic cells were used from the published manuscript [32]. Quantification was done at 12 different timepoints (*t*=2*h* *r*,12*h* *r*,1*d*,2*d*,3*d*,4*d*,8*d*,11*d*,15*d*,18*d*,22*d*,32*d*, assuming *t*=0 at the moment of BrdU injection) (Table 2). 2) Single BrdU pulse-and-chase was used to quantify BrdU+ NSCs and ANPs using GFAP and GFP immunostaining to differentiate between them. BrdU+ GFP+ NSCs were identified by their localization in the SGZ, radial GFAP+ process, and fine eGFP+ terminal arborizations in the molecular layer. BrdU+ GFP+ ANPs were identified by their localization in the SGZ, round morphology with no processes, and no GFAP staining. Quantification was done at *t*=2*h* *r*,1*d*,2*d*,4*d*, and 8*d* (Table 3). 3) Single BrdU pulse-and-chase was used to quantify NB, IN, and GC using DCX and NeuN immunostaining and morphology. Newborn NBs were BrdU+ DCX+ NeuN- or NeuN+ round cells with small processes. Newborn GC were BrdU+ DCX- Neu+ mature neurons within the granule cell layer. Quantification was done at *t*=1*d*,2*d*,4*d*,8*d*,15*d* (Table 3). In all experiments, mice were 1 month old at the time of BrdU injection (*N* = 2-5 mice per timepoint).

**Table 2:** Total BrdU+ cell count and BrdU+ apoptotic cell count

Time (days)	*n*	Total BrdU+ cells	BrdU+ apoptotic cells
0.08 (2hr)	3	2690 (320)	0 (0)
0.5 (12h)	2	4157 (784)	0 (0)
1	4	5392 (557)	40 (18)
2	5	5803 (138)	121 (33)
3	3	4781 (344)	48 (25)
4	5	4186 (201)	23 (14)
8	6	3518 (307)	10 (11)
11	3	2427 (202)	0 (0)
15	4	1342 (185)	33 (13)
18	3	1233 (302)	0 (0)
22	4	752 (53)	0 (0)
32	3	950 (234)	13 (16)

*n* is the sample size. Cell numbers are represented as the mean and standard error of the mean (sem) (Sierra et al., 2010)

**Table 3:** Estimated proportion of BrdU+ cells of each type

Time (days)	Experiment 1			Experiment 2		
	*n*	NSC	ANP	*n*	NB	GC
0.08 (2hr)	4	11.16 (2.14)	85.07 (3.62)	-	-	-
0.5 (12h)	-	-	-	-	-	-
1	4	5.68 (0.57)	60.14 (2.59)	3	51.94 (7.25)	0.2 (0.24)
2	5	3.29 (0.79)	42.31 (4.81)	2	76.42 (3.24)	0.32 (0.46)
3	-	-	-	-	-	-
4	5	2.53 (0.69)	20.37 (0.85)	3	95.06 (1.12)	1.52 (0.51)
8	5	0 (0)	4.87 (1.38)	3	96.24 (0.76)	2.48 (0.31)
11	-	-	-	-	-	-
15	-	-	-	2	86.61 (1.26)	4.72 (0.05)
18	-	-	-	-	-	-
22	-	-	-	-	-	-
32	-	-	-	3	14.86 (3.62)	77.34 (6.81)

*n* is the sample size, “-” means no available data. Two groups of animals (all 1 month old) were used for experiments. Cell numbers are represented as the mean and standard error of the mean (sem) in proportion (×100) of cells of each type

Given the estimated number of cells during the S-phase in each stage at the beginning of BrdU injection, we may calculate the number of labeled cells of each type at any moment by Eq. (1). However, solving it in analytical form is cumbersome. An approach alternativee to computationally producing the BrdU labeling curves is the event-based simulation. Assuming that we have computed the numbers of cells in different stages at the moment of BrdU injection (*t*=0), we trace the fate of labeled cells at unit time points by recording their behaviors. Briefly, a series of random numbers are generated for the random times for which labeled cells stay in particular stages and the probability of the cells transiting to the next stage, until the cells enter apoptosis or become a matured neuron. Beginning to trace the entire process from *t*=0, we reproduce the labeling curves *in silico*by accumulating the fates of all labeled cells up to particular moments (e.g. times of measurements in experiments).

**Fig. 5:**
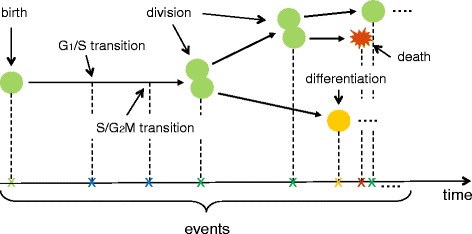
Simulation scheme of the dynamic of a neuroprogenitor cell. The event-based simulation traces the fate of a newborn, BrdU-labeled cell (green icon). Assuming that we have computed the numbers of cells in different stages of the neurogenic cascade at the moment of BrdU injection (t = 0), we trace the fate of BrdU labeled cells at unit time points by recording their behaviors. Briefly, a series of random numbers are generated for the random times for which labeled cells stay in particular stages and for the probability of the cells transiting to the next stage, until the cells die (red icon) or become a mature neuron (yellow icon)

A simulation program carrying out tasks outlined above has been written in the Python programming language. This program computes distribution of initial cell population and generates BrdU labeling curves. Figure 5 illustrates the event-based simulation scheme of the dynamics of a neuroprogenitor cell.

#### Parameter search and goodness-of-fit

To discover parameter combinations that can best fit the experimental data, we adapted a genetic algorithm as the searching heuristic since the parameter space is too complex to be searched by enumeration of all possible combinations. A genetic algorithm is a searching heuristic that mimics the process of natural evolution. It is used to generate optimized solutions to search problems in complex non-linear systems. Each parameter range is encoded by a bit vector of length *a*, yielding 2^*a*^ possible values. An initial pseudo “population” was created by setting *X* randomly chosen parameter combinations as *X* “individuals”. The value of each modeling parameter in any “individual” has been converted to the binary format to become a 0-1 sequence. During the search, any parameter set is evaluated by computing variance weighted least square error $\sum \frac {(E-S)^{2}}{\sigma ^{2}}$ to test how well the simulated results fit the experimental labeling curves. *E* and *σ* ^2^ are mean and variance of experimental data at a given time point, whereas *S* is the corresponded simulation result. $\sum $ sums over all available time points of measurements. The list of model parameters is shown in Table 1. Some of them can be experimentally estimable, whereas most are not observable.

## Results

## Cell proportions

At time points *t= 1, 2, 4*and *8* days, the sum of observed proportions of all cell types (NSCs, ANPs, NBs, GCs and apoptotic cells) is greater than 1 (Tables 2 and 3). This discrepancy is due to the non-specific labeling or identification of cells during transitions from ANPs to NBs. Cells that are in the intermediate stage can be labeled by both ANP and NB markers. Thus, we assume that the estimated proportions on NSCs and GCs are realistic, while those of ANPs and NBs at intermediate time points are inflated due to non-specific labeling. Those quantifications need to be adjusted. At early (2hr) or late time points (15 and 32 days), we retained the original data since at those time points the labeled cells are NSCs and GCs. At all other timepoints, the labeled cells are either ANPs or NBs, exclusively.

For any time point *t* (*t* = 1, 2, 4 or 8 days), we adjusted the sum of proportions of all types of BrdU+ cells (NSCs, ANPs, NBs, GCs and apoptotic cells) to be equal to 1 (impact of BrdU+ astrocytes is negligible since the proportion of BrdU+ astrocytes is very small when *t*≤8 days [41]). The proportion of the excessive amount of cells (denoted by *d*_*t*_) between ANPs and NBs is equal to the sum of the observed proportions minus 1. We denote *α*_*t*_ to be the ratio defined so that *α*_*t*_ *d*_*t*_ is the proportion of double labeled cells that belong to ANPs, whereas (1−*α*_*t*_)*d*_*t*_ belong to NBs.

Let **X**=(*X*_1_,*X*_2_)^*T*^ and **X** ^′^=(*X*1′,*X*2′)^*T*^ as two random vectors to represent the proportions of ANPs and NBs before and after transformation, respectively. Therefore, we obtain

$$\begin{aligned} X_{1}' & = X_{1}-\left(1-\alpha_{t}\right)\left(X_{1}+X_{2}-d_{t}\right)\\ &=\alpha_{t}X_{1}+\left(\alpha_{t}-1\right)X_{2}+\left(1-\alpha_{t}\right)d_{t}\\ X_{2}' & = X_{2}-\alpha_{t}\left(X_{1}+X_{2}-d_{t}\right)\\ &=-\alpha_{t}X_{1}+\left(1-\alpha_{t}\right)X_{2}+\alpha_{t}d_{t} \end{aligned} $$

and

$$\mathbf{X}'=\mathbf{A}\mathbf{X}+\mathbf{B} $$

where $\mathbf {A}=\left (\begin {array}{cc} \alpha _{t} & \alpha _{t}-1\\ -\alpha _{t} & 1-\alpha _{t} \end {array}\right)$, $\mathbf {B}=\left (\begin {array}{c} (1-\alpha _{t})d\\ \alpha _{t} \end {array}\right)$.

Thus, we can compute the mean and variance of *X* ^′^ as

$$ \begin{aligned} E[\mathbf{X}'] & = \left(\alpha E[X_{1}]+(\alpha_{t}-1)E[X_{2}] \right.\\ & \quad + \left. (1-\alpha_{t})d_{t},-\alpha_{t}E[X_{1}]+(1-\alpha_{t})E[X_{2}]+\alpha_{t}d_{t}\right) \end{aligned} $$

$${{}\begin{aligned} \Sigma[\mathbf{X'}] = \left(\begin{array}{cc} V[\!X_{1}'] & COV[X_{1}',X_{2}']\\ COV[X_{1}',X_{2}'] & V[X_{2}'] \end{array}\right) =\mathbf{A^{T}}\mathbf{\Sigma[}\mathbf{X}\mathbf{]A} \end{aligned}} $$

where *Σ*[**X** ^′^] and *Σ*[**X**] are covariance matrices of **X** ^′^ and **X** with *Σ*[**X**]=(*V*[*X*_1_],*V*[*X*_2_]) by assuming that *C* *O* *V*[*X*_1_,*X*_2_]=0. Using the equations above, we calculated the adjusted estimates of means and sems of ANP and NB cell proportions (Table 4). Note that for any interval time point *t* (*t*=1*d*,2*d*,4*d*,8*d*), *α*_*t*_ is assumed to be 1/2 since there is no prior knowledge about it.

**Table 4:** Re-proportioned data of estimated proportions of BrdU+ cell of each type

Time (days)	Experiment 1			Experiment 2		
	*n*	NSC	ANP	*n*	NB	GC
0.08 (2hr)	4	11.16 (2.14)	85.07 (3.62)	-	-	-
0.5 (12h)	-	-	-	-	-	-
1	4	5.68 (0.57)	**50.79 (3.23)**	3	**42.59 (3.95)**	0.2 (0.24)
2	5	3.29 (0.79)	**30.29 (2.54)**	2	**64.41 (5.08)**	0.32 (0.46)
3	-	-	-	-	-	-
4	5	2.53 (0.69)	**10.35 (0.58)**	3	**85.05 (0.82)**	1.52 (0.51)
8	5	0 (0)	**1.74 (0.74)**	3	**93.11 (1.05)**	2.48 (0.31)
11	-	-	-	-	-	-
15	-	-	-	2	86.61 (1.26)	4.72 (0.05)
18	-	-	-	-	-	-
22	-	-	-	-	-	-
32	-	-	-	3	14.86 (3.62)	77.34 (6.81)

*n* is the sample size, “-” means no available data. Numbers in bold are adjusted values of proportions (×100). Cell numbers are represented as the mean and standard error of the mean (sem) of the proportion (×100) of each type of cells

### Transforming cell proportions to cell counts

For the optimization algorithm, the total number of BrdU+ cells and the estimated number of BrdU+ cells of each specific type are required to evaluate the goodness-of-fit. Non-zero data points expressed as proportions (random variables; Table 3) were transformed back to cell counts with re-calculated means and variances.

For any time point *t*, we assumed that the count of total number of BrdU+ cells in an animal is a normally distributed random variable $Y\sim N\left (\mu _{Y},\sigma _{Y}^{2}\right)$. Thus, for a size of *n*_*Y*_ samples, we obtain that $\hat {\mu _{Y}}=\overline {Y}$ and $\hat {\sigma _{Y}^{2}}=S_{Y}^{2}$ (data from Table 1 were used to estimate $\hat {\mu _{Y}}$ and $\hat {\sigma _{Y}^{2}}$).

For any specific cell type *i* (e.g. ANPs), if we denote *X* as the number of BrdU+ type *i* cells at time *t*, we have

$$X|Y,P\sim\text{binomial}(Y,P) $$

where we assume that the observed proportion of type *i* cell, *P*, is Gaussian distributed, s.t. $P\sim N\left (\mu _{P},\sigma _{P}^{2}\right)$. For a size of *n*_*P*_ samples, $\hat {\mu _{P}}=\overline {P}$ and $\hat {\sigma _{P}^{2}}=S_{P}^{2}$ $\left ({\vphantom {\hat {\sigma _{P}^{2}}}}\hat {\mu _{P}}\right.$ and $\hat {\sigma _{P}^{2}}$ are estimated from data shown in Table 4).

Assuming that *Y* and *P* are independent random variables, we obtain that

$$E[X]=E[E[X|Y,P]]=E[YP]=E[Y]E[P] $$

and

$$\begin{array}{@{}rcl@{}} V[X] & = & V[E[X|Y,P]]+E[V[X|Y,P]]\\ & = & V[YP]+E[YP(1-P)]\\ & = & V[YP]+E[Y]E[P]-E[Y]E[P^{2}]\\ & = & V[YP]+E[Y]E[P]-E[Y](V[P]+E[P]^{2}) \end{array} $$

Since

$$V[YP]=E[Y]^{2}V[P]+E[P]^{2}V[Y]+V[Y]V[P] $$

we obtain

$$\begin{aligned} V[X]&=V[P]\left(E[Y]^{2}+V[Y]-E[Y]\right)\\ &\quad+E[P]^{2}(V[Y]-E[Y])+E[Y]E[P] \end{aligned} $$

Therefore, we estimate the mean and variance of the number of BrdU+ labeled cells of any type *i* (*E*[*X*] and *V*[*X*]) by equations below:

$$\begin{array}{@{}rcl@{}} E[X] & = & \overline{Y}\times\overline{P}\\ V[X] & = & S_{p}^{2}\left(\overline{Y}^{2}+S_{Y}^{2}-\overline{Y}\right)+\overline{P}^{2}\left(S_{Y}^{2}-\overline{Y}\right)+\overline{Y}\times\overline{P} \end{array} $$

A summary of data in estimated cell numbers is shown in Table 5.

**Table 5:** Summary of estimated cell counts in each type

Time (days)	total	Apop	NSC	ANP	NB	GC
0.08 (2hr)	2690 (320)	0 (0)	300 (81)	2288 (298)	-	-
0.5 (12h)	4157 (784)	0 (0)	-	-	-	-
1	5392 (557)	40 (18)	306 (46)	2738 (334)	2296 (297)	11 (11)
2	5803 (138)	121 (33)	191 (47)	1758 (154)	3738 (173)	19 (14)
3	4781 (344)	48 (25)	-	-	-	-
4	4186 (201)	23 (14)	106 (30)	433 (34)	3560 (173)	64 (16)
8	3518 (307)	10 (11)	0 (0)	61 (25)	3276 (287)	87 (11)
11	2427 (202)	0 (0)	-	-	-	-
15	1342 (185)	33 (13)	-	-	1162 (160)	63 (10)
18	1233 (302)	0 (0)	-	-	-	-
22	752 (53)	0 (0)	-	-	-	-
32	950 (234)	13 (16)	-	-	141 (51)	735 (194)

“-” : no available data. Cell numbers are represented as the mean and standard error of the mean (sem) for estimated numbers of different types of cells

### Initial distribution of cell population

Before deriving the model to simulate BrdU labeling curves, we focused on the quantification of the cell populations at the moment of the BrdU injection and aimed to obtain the distribution of cell populations at *t*=0.

In matrix *M* (Eq. (1)), the fundamental solution of the model *M*_*ij*_ as a function of time, allows finding the number of cells at time *t* in compartment *j*, given that the population was seeded by a single cell in compartment *i*. However, under physiological conditions, the system is fed by a steady influx of freshly activated ANPs. Under such assumption, the number of of cells of each cell type at time *t* is given by

3 $$ \widetilde{M}(t)=\lambda\sum_{k=0}^{\infty}[T(t)m]^{*k}*\int_{0}^{t}[(I-T(\tau)]d\tau  $$

Since the system is fed only by newborn ANPs at their first division in *G*_1_-phase, only the top row of matrix $\widetilde {M}(t)$, denoted by $\widetilde {M}^{(1)}(t)$, is required. In labeling experiments, we treat 1 month old mice with BrdU injections. We assume that the snapshot of the neurogenic cell population in the animal’s brain is under a “steady state” at the moment of injection. Thus, if we tend with *t* to infinity in the Eq. (3), we can obtain a stationary distribution of cell numbers in the snapshot, which yields numbers of cells of different types, given by

$$\begin{aligned} \pi&=\widetilde{M}^{(1)}(\infty)\\ &=\left({\lim}_{t\rightarrow\infty}\lambda\sum\limits_{k=0}^{\infty}[T(t)m]^{*k}*\int_{0}^{t}[(I-T(\tau)]d\tau\right)^{(1)}\\ &=\left(\lambda(I-m)^{-1}E[T]\right)^{(1)} \end{aligned} $$

where *π* is the vector of numbers of cells in all modeling compartments (see Matrix Eq. (2) for the description of modeling compartments), diagonal matrix *E*[*T*] has entries equal to expected duration times in all modeling compartments, (·)^−1^ denotes the inverse of a matrix, (·)^(1)^ denotes the top row of the matrix and *λ*,*T*,*m*,*I*,and∗ are as previously defined. Although in the long run the intensity parameter *λ* declines as the animal gets older, we assume that in a short period of time, within which the snapshot of the BrdU injection occurs, the influx rate of NSCs to newborn ANPs is constant.

BrdU labels all cells that are in S-phase, thus we know how many cells are labeled at the moment of injection, which is equal to *π*_*s*_, where subscript *s* stands for the compartment or a set of compartments which represents the cells in the S-phase. Under the assumption that in vivo descendants of labeled cells remain labeled (BrdU dilution is negligible), the BrdU pulse labeling curve of the number of labeled cells at a given time is equal to *π*_*s*_ *M*(*t*). This expression is technically true only under the assumption that labeled cells concentrated at the beginning of the S-phase, which does not make much difference for times longer than the joint duration of *S* and *G*_2_ *M*. In a real situation, the time remaining for each cell in S-phase at any moment is a random variable. Additionally, the *k*-fold convolution of matrix (*T* *m*) in the matrix *M*(*t*) (Eq. (1)) becomes analytically too complicated as *k* increases. Therefore, as explained before, instead of obtaining the time-course labeling curve analytically, we decided to generate it by simulation in a more convenient and straightforward manner.

### Simulation of BrdU labeling curves and parameter search

To obtain the set of model parameters that yields best fit to the experimental observation of BrdU pulse-and-chase labeling curves, we applied the genetic algorithm as the search heuristic in the simulation program. Table 6 lists model parameters and their assigned ranges of possible values. For example, the maximum number of ANP divisions ranges from 2 to 8, renewal probability of ANPs ranges from 0 to 1, expected duration of ANP S-phase ranges from 5 to 12 hr, shape parameter of distribution of ANP S-phase duration ranges from 5 to 40, minimum duration of ANP S-phase ranges from 1 to 4 and apoptotic rate for ANP S-phase can range from 0 to 0.99.

**Table 6:** List of model parameters and ranges of possible values

Parameter	Range of possible values
Minimum number of ANP divisions, *m* *i* *n*_*ANP*_	1,2,3
Maximum number of ANP divisions, *m* *a* *x*_*ANP*_	2,...,8
Renewal probability of ANP, *p*_*ANP*_	{0,0.1,...,0.99,1}
Distribution coefficients of ANP *G*_1_-phase duration, $T_{G_{1}-ANP}$	6,...,20*h*r; {2,...,16}; {2,...,5}*h* *r*
Distribution coefficients of ANP S-phase duration, *T*_*S*−*ANP*_	{5,...,12}*h* *r*; {5,...,40}; {1,...,4}*h* *r*
Distribution coefficients of ANP *G*_2_ *M*-phase duration, $T_{G_{2}M-ANP}$	{1,...,4}*h* *r*; {5,...,20}; {0,...,0.75}*h* *r*
Distribution coefficients of ANP-NB stage duration^1^, *T*_*ANP*−*NB*_	{4,...,64}*h* *r*; {2,...,16}; {0,...,3}*h* *r*
Distribution coefficients of ANP-Apop stage duration^2^, *T*_*ANP*−*Apop*_	{4,...,64}*h* *r*; {2,...,16}; {0,...,3}*h* *r*
Distribution coefficients of NB duration, *T*_*NB*_	{120,...,430}*h* *r*; {2,...,16}; {10,...,80}*h* *r*
Distribution coefficients of Apoptotic cell duration, *T*_*Apop*_	{0.4,...,3}*h* *r*; {2,...,16}; {0,...,0.3}*h* *r*
Cell death rate of ANP *G*_1_-phase, $d_{G_{1}}$	{0,0.1,...,0.98,0.99}
Cell death rate of ANP S-phase, *d*_*S*_	{0,0.1,...,0.98,0.99}
Cell death rate of ANP *G*_2_ *M*-phase, $d_{G_{2}M}$	{0,0.1,...,0.98,0.99}
Cell death rate of non-proliferating ANP, *d*_*ANP*_	{0,0.1,...,0.98,0.99}
Cell death rate of NB, *d*_*NB*_	{0,0.1,...,0.98,0.99}
Minimum number of NSC divisions, *m* *i* *n*_*QNP*_	{1,2,3}
Maximum number of NSC divisions, *m* *a* *x*_*QNP*_	{2,...,6}
Renewal probability of NSC, *p*_*QNP*_	{0,0.1,...,0.99,1}
Distribution coefficients of NSC *G*_1_-phase duration, $T_{G_{1}-QNP}$	{8,...,36}*h* *r*; {2,...,16}; {2,...,5}*h* *r*
Distribution coefficients of NSC S-phase duration, *T*_*S*−*QNP*_	{5,...,12}*h* *r*; {5,...,40}; {1,...,4}*h* *r*
Distribution coefficients of NSC *G*_2_ *M*-phase duration, $T_{G_{2}M-QNP}$	{1,...,4}*h* *r*; {5,...,20}; {0,...,0.75}*h* *r*

^1^If a non-proliferative ANP is determined to differentiate to a NB, it enters ANP-NB stage,

^2^otherwise it enters ANP-Apop stage before undergoing apoptosis. Since the cell duration (transit time) is modeled by a shifted gamma distribution the duration distribution parameter for any cell type *i*, *T*_*i*_, consists of 3 coefficients that are expected duration, shape parameter of the gamma distribution and the minimum duration (shift value). A range of values for each of these three coefficients has been provided

**Fig. 6:**
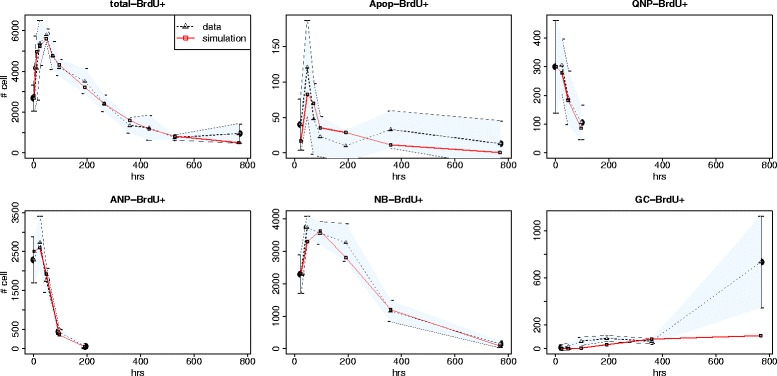
Fitting model to experimental BrdU labeling curves - data vs simulation results. Simulation results (red solid lines) that best fit the data (black dashed lines) of all available measurements from running the genetic algorithm are presented for all investigated cell types. Total BrdU+ = total number of BrdU+ cells, Apop-BrdU+ = number of BrdU labeled apoptotic cells, QNP-BrdU+=number of BrdU labeled NSCs, ANP-BrdU+ = number of BrdU labeled ANPs, NB-BrdU+ and GC-BrdU+ = numbers of BrdU labeled neuroblasts and granule cells, respectively. On each plot, the shaded area depicts the region that is upper and lower bounded by the average cell counts ± 2 SEM

**Fig. 7:**
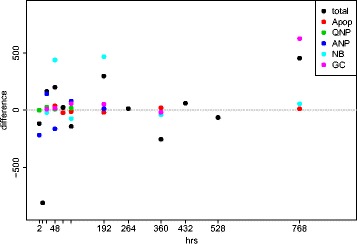
Residuals plot shows the distribution of differences (experimental data – simulated results) on all non-zero measurements. The residuals were equally distributed along x axis and showed no systematic trend, suggesting that the model fit is good

Simulation results of BrdU labeling curves that best fit our data are illustrated in Fig. 6. Parameter values that yield such fits were obtained by the genetic algorithm, which is implemented for the purpose of searching the parameter space and optimizing the goodness-of-fit function. BrdU pulse labeling experiments have provided us with 40 non-trivial (non-zero) independently measured experimental data points. Nineteen model parameters are varied during the parameter search. Figure 7 demonstrates that the residuals were equally distributed along *x* axis and showed no systematic trend, which suggests that the model fit is good.

**Fig. 8:**
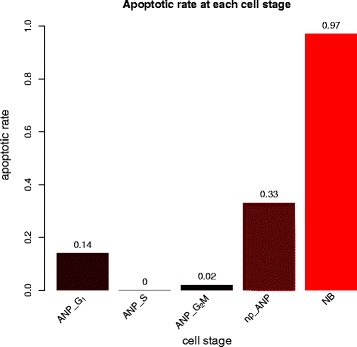
Estimated apoptosis rate at each cell stage that yields best fit to data. The bar graphs show estimated apoptotic rates at each cell stage through the early stages of the hippocampal neurogenic cascade in a 1-month old mouse. The apoptosis is highest among neuroblasts (NBs), followed by non-proliferating ANPs (np-ANP). This is in agreement with experimental data, indicating that model fit is good

Among all model parameters, apoptotic rates are the most critical ones since they have not been estimated in prior early-stage hippocampal neurogenesis studies. Based on our model and simulation results at each cell state during early stages of hippocampal neurogenesis in 1-month-old mice, we estimate that the apoptosis rates are low in proliferating ANPs whereas once ANPs become non-proliferating, about one third of them undergo apoptosis (Fig. 8). During the NB stage, apoptosis reaches maximum. A vast majority of the NBs die (97% undergo apoptosis) and only a few of them (estimated about 3%) will differentiate into the GCs. NSCs do not undergo apoptosis [32] and once a NSC is activated, it undergoes a number of asymmetric divisions after which it eventually becomes an astrocyte.

### Prediction of dynamics of neurogenesis under reduced apoptosis

**Fig. 9:**
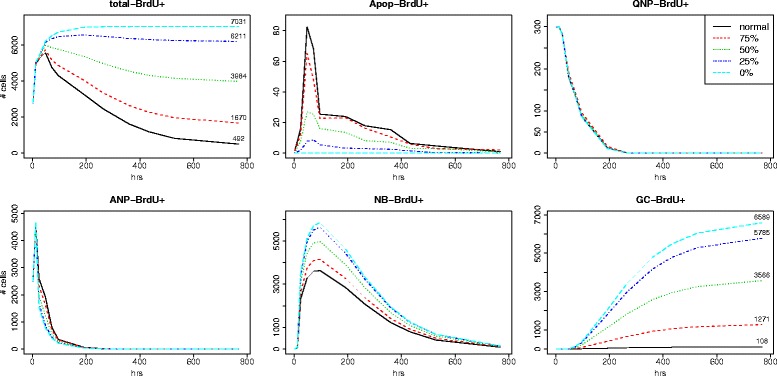
Prediction of dynamics of neurogenesis under reduced apoptosis. Effect of reducing apoptosis on simulated labeling curves of different types of cells over the time course of 32 days. Black line = normal apoptotic rates; red = apoptotic rates reduced to 75% of the normal rates; green = apoptotic rates reduced to 50% of the normal rates; blue = apoptotic rates reduced to 25% of the normal rates; light blue = apoptotic rates reduced to 0%

Taking values of model parameters from data fitting results, we carried out additional simulations to predict the overall changes in the BrdU labeling curves by inhibiting apoptosis (reducing apoptotic rates). While apoptotic rates at all cell stages were consistently reduced by a hypothetical amount (25%, 50%, 75% or 100%) all other model parameters remain unchanged. From predicted BrdU labeling curves depicted in Fig. 9, we observed that at the end of 32 days, the total number of BrdU+ cells and the number of BrdU labeled granule cells increased 3.4 and 11.5 fold, respectively, when apoptotic rates were reduced by 25% only. These numbers continue to increase sharply if apoptosis can be reduced even further. Under the extreme scenario, when apoptosis can be completely inhibited, the simulation results indicate that 14.3 times more of total BrdU+ cells and 61.0 times more of BrdU+ GCs are expected as the net outcome of neurogenesis, compared with the case when physiologic apoptotic rates are employed. Our study thus indicates that reducing apoptosis in any amount substantially increases adult hippocampal neurogenesis.

## Discussion

In this study, we developed a computational model that as accurately as possible reflects the neurogenic cascade and specifically, apoptosis. Our goal was to estimate apoptotic rates at each stage of the neurogenic cascade, the distribution of cell type duration – including apoptosis, and the renewal probability of ANPs. We reasoned that these parameters were most important to design targeted experimentation to improve survival of newborn cells and net outcome of hippocampal neurogenesis. Since there is unavoidably a large amount of model parameters providing challenges and obstacles to unbiased estimation, we employed immunohistochemistry and statistical computation approaches to combine experimental and computational data. Furthermore, we computationally estimated experimentally unobservable parameters, such as the probability of ANP to proliferate and the rates of cells at different stages undergoing apoptosis. Our model indicates that apoptosis is low in the ANP stage and high in the NB stage. Regardless of origin, apoptotic cells have a short life, estimated to be around 1.4hrs. ANPs are predicted to divide 1–4 times; however, their renewal probability is low, at 0.1. Finally, the NB stage has the largest variance of the transit time. None of the estimates could be derived experimentally, and thus, our computational model represents a foundation upon which we can design novel biological experiments to increase neurogenesis based on targeted action on ANPs and newborn cell survival. Encinas et al. (2011) carried out labeling experiments (both single and cumulative labeling) to study adult hippocampal neurogenesis. They modeled neurogenic cascade similarly as we do, although they used 2 month old mice whereas we used 1 month old ones. While Encinas et al. (2011) determined division and duration related parameters, they did not infer any information on apoptosis. Their results were calculated from inferring the decay rate of each type of cell over a long period of time (800 days). In comparison, the parameter values that yield best fit in our study were comparable with respect to expected cell durations (Table 7). In addition, our model and simulation approach are able to provide estimates on apoptotic rates, minimum durations, shapes of duration distributions, and number of NSC and ANP divisions. More recent works [42, 43] investigates the regulatory mechanisms of neurogenesis, based on knockout experiments, which modify the dynamic behaviour of this process. Evaluating these knockout is a non-trivial task owing to the complicated nature of the hippocampal neurogenic niche. Unlike the model proposed herein, they model neurogenesis as a multicompartmental system of ordinary differential equations based on experimental data. To analyse the results of knockout experiments, they investigated how changes of cell properties, based on cells labelled by the cell division marker BrdU. Among other, they found that changing cell proliferation rates or the fraction of self-renewal, may result in multiple time phases in the response of the system, such as an initial increase in cell counts followed by a decrease. Because of different experimental setup and modeling framwork used, these results are not directly comparable to ours. One of the obstacles is the difficulty in observing and recording the fates of the individual cells in vivo.

**Table 7:** Parameter estimates that yield best fit and comparison with estimates in literature

Parameter	Value	Estimate of
		Encinas et al.
Minimum number of ANP divisions	1	-
Maximum number of ANP divisions	4	-
Renewal probability of ANP	0.1	-
Expected number of ANP divisions	1.17	2
Expected ANP *G*_1_-phase duration	12hr	-
Minimum ANP *G*_1_-phase duration	3hr	-
Expected ANP S-phase duration	12hr	12hr
Minimum ANP S-phase duration	4hr	-
Expected ANP *G*_2_ *M*-phase duration	1hr	2hr
Minimum ANP *G*_2_ *M*-phase duration	0.5hr	-
Expected ANP-NB^1^ duration	12hr	30hr
Minimum ANP-NB duration	3hr	-
Expected ANP-Apop^2^ duration	48hr	-
Minimum ANP-Apop duration	2hr	-
Expected NB duration	260hr	60hr & 306hr^3^
Shape parameter of NB duration distribution	2	-
Minimum NB duration	20hr	-
Expected apoptotic cell duration	1.4hr	-
Cell death rate of ANP *G*_1_-phase	0.14	-
Cell death rate of ANP S-phase	0	-
Cell death rate of ANP *G*_2_ *M*-phase	0.02	-
Cell death rate of nonproliferating ANP	0.33	-
Cell death rate of NB	0.97	-
Minimum number of NSC divisions	2	-
Maximum number of NSC divisions	5	-
Renewal probability of NSC	0.57	-
Expected number of NSC divisions	3.57	3
Expected NSC *G*_1_-phase duration	28hr	-
Expected NSC S-phase duration	11hr	8hr
Expected NSC *G*_2_ *M*-phase duration	3hr	2hr
Expected NSC duration	42hr	28hr, 28hr
		and 52hr^4^

^1^ANP-NB is the transition stage between ANP and NB

^2^ANP-Apop is the transition stage between ANP and apoptotic cells

^3^NB durations for *t*<100*h* *r* and *t*>100*h* *r*, respectively

^4^Expected durations of the first, second and third divisions

## Conclusion

In sum, our computational model of the adult neurogenesis provides new information on the early stages of this phenomenon. It is our hope that the estimates of the properties of ANPs, NBs, and apoptotic cells will guide biological investigations and development of better experimental tools to utilize this unique process for the benefit of human health.

## Abbreviations

ANP: Amplifying neuroprogenitor
Apop: Apoptotic cell
BrdU: Bromodeoxyuridine
BSA: Bovine serum albumin
CFP: Cyan fluorescent protein
DCX: Doublecortin
GC: Granule cell
GFAP: Glial fibrilary acidic protein
GFP: Green fluorescent protein
IN: Immature neuron
NB: Neuroblast
NPCs: Neural stem and progenitor cells
NSC: Neural stem cell
PBS: Phosphate-buffered saline
PFA: Paraformaldehyde
SGZ: Sub-granular zone
